# Distributed lag effects and vulnerable groups of floods on bacillary dysentery in Huaihua, China

**DOI:** 10.1038/srep29456

**Published:** 2016-07-18

**Authors:** Zhi-Dong Liu, Jing Li, Ying Zhang, Guo-Yong Ding, Xin Xu, Lu Gao, Xue-Na Liu, Qi-Yong Liu, Bao-Fa Jiang

**Affiliations:** 1 Department of Epidemiology, School of Public Health, Shandong University, Jinan, Shandong Province, People’s Republic of China; 2 Shandong University Climate Change and Health Center, Jinan, Shandong Province, People’s Republic of China; 3 School of Public Health, China Studies Centre, The University of Sydney, New South Wales, Australia; 4 Department of Epidemiology, School of Public Health, Taishan Medical College, Taian, Shandong Province, People’s Republic of China; 5 State Key Laboratory for Infectious Disease Prevention and Control, National Institute for Communicable Disease Control and Prevention, China CDC, Beijing 102206, P. R. China

## Abstract

Understanding the potential links between floods and bacillary dysentery in China is important to develop appropriate intervention programs after floods. This study aimed to explore the distributed lag effects of floods on bacillary dysentery and to identify the vulnerable groups in Huaihua, China. Weekly number of bacillary dysentery cases from 2005–2011 were obtained during flood season. Flood data and meteorological data over the same period were obtained from the China Meteorological Data Sharing Service System. To examine the distributed lag effects, a generalized linear mixed model combined with a distributed lag non-linear model were developed to assess the relationship between floods and bacillary dysentery. A total of 3,709 cases of bacillary dysentery were notified over the study period. The effects of floods on bacillary dysentery continued for approximately 3 weeks with a cumulative risk ratio equal to 1.52 (95% CI: 1.08–2.12). The risks of bacillary dysentery were higher in females, farmers and people aged 15–64 years old. This study suggests floods have increased the risk of bacillary dysentery with 3 weeks’ effects, especially for the vulnerable groups identified. Public health programs should be taken to prevent and control a potential risk of bacillary dysentery after floods.

Floods are the most common type of natural disaster globally. On average, floods and other hydrological events had accounted for over 50% of the natural disasters between 2001 and 2010 in the world^1^. Floods are expected to increase in frequency and intensity due to rising sea levels and more frequent and extreme precipitation events^2^. Hunan, located in the basin of Yangtze River, is a flood-prone province in China. Persistent and heavy rainfall caused several floods from 2005–2011 in Huaihua City of Hunan Province^3^.

Bacillary dysentery, a diarrheal disease caused by ingestion of water or food contaminated by different species of *Shigella* bacteria, remains a major public health problem in some developing countries^4^. In China, 237,930 new cases of bacillary dysentery were reported in 2011, which ranked fourth among the national notifiable diseases from the Chinese National Notifiable Disease Report. Hunan is one of the most seriously affected provinces in the Yangtze River Region. Huaihua city, which located in the west of Hunan, had 5,414 cases from 2005–2011^5^.

The association between floods and bacillary dysentery is not clear. Some epidemiologic evidence suggests that floods are positively associated with diarrheal diseases, such as dysentery, cholera, and paratyphoid fever^6^,^7^. During the flood in the Midwestern United States in 2001, Wade *et al*. found 1.29 times increase in the incidence of gastrointestinal symptoms^8^. A significant positive association between floods and diarrhea was reported by Heller *et al*. in Brazil^9^. A study from Henan of China revealed that floods were significantly associated with dysentery^10^. However, a study from Mozambique revealed that there was no outbreak of dysentery after the flooding in 2000^11^. Another study also found no clear evidence of excess mortality or diarrhea risk during or after flooding after controlling for pre-flood rate differences and seasonality^12^. More research is needed to elucidate the potential risk of bacillary dysentery related to floods. Research linking floods to bacillary dysentery in China is limited. The effect of the floods on bacillary dysentery in Huaihua remains unknown. This study aimed to investigate the distributed lag effects on bacillary dysentery related to floods and identify the vulnerable groups. Results will contribute to a better understanding of the health impacts of floods and provide more evidence to support decision-making for the prevention and control of bacillary dysentery after floods.

## Results

### Description of the disease and meteorological data

**Figure 1:**
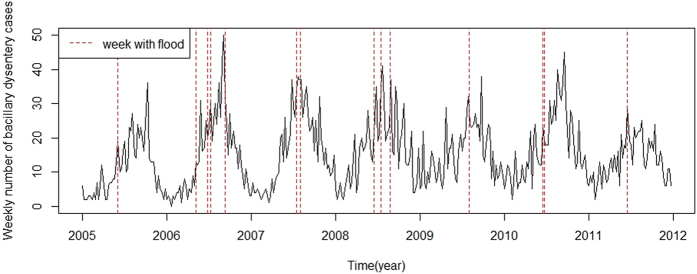
The weekly distribution of bacillary dysentery cases during study period from 2005 to 2011 in Huaihua, China. Weeks with flood is indicated as a dashed line.

**Figure 2:**
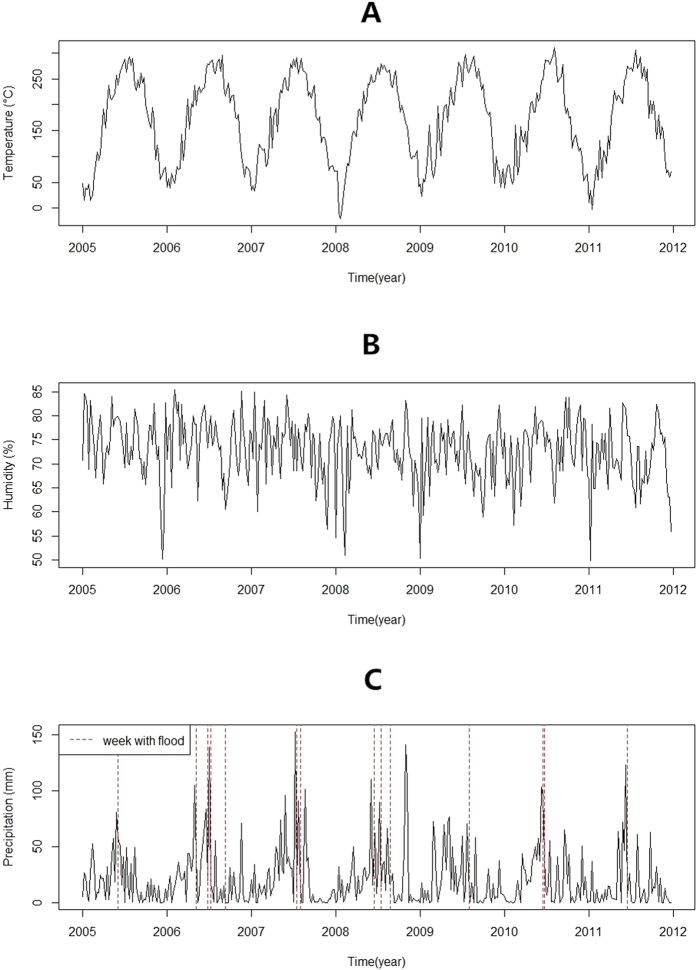
Weekly mean temperature (**A**) weekly mean relative humidity (**B**) and weekly cumulative precipitation (**C**) during study period from 2005–2011 in Huaihua. Weeks with flood is indicated as a dashed line in (**C**).

A total of 3,709 cases of bacillary dysentery were notified in the study area over the study period. Table 1 presents the descriptive statistics for weekly bacillary dysentery cases in different categories from 2005–2011 in the flood season of Huaihua. There were more cases in male living in rural areas. Most of the bacillary dysentery cases were children and farmers. Patients aged 0–4 and 15–64 accounted for approximately 76% of all bacillary dysentery cases. Figures 1 and 2 show the time-series distribution of weekly bacillary dysentery cases and weekly mean temperature, weekly mean relative humidity and weekly cumulative precipitation in the flood season from 2005–2011. The average number of weekly bacillary dysentery cases was 20 (range: 2–50). The average values of weekly mean temperature, weekly mean relative humidity and weekly cumulative precipitation were 24 °C (range: 10–31 °C), 73% (range: 58–84%) and 28 mm (range: 0–153 mm), respectively. Fourteen flood events were recorded during the study period.

### Association between floods and bacillary dysentery

**Figure 3:**
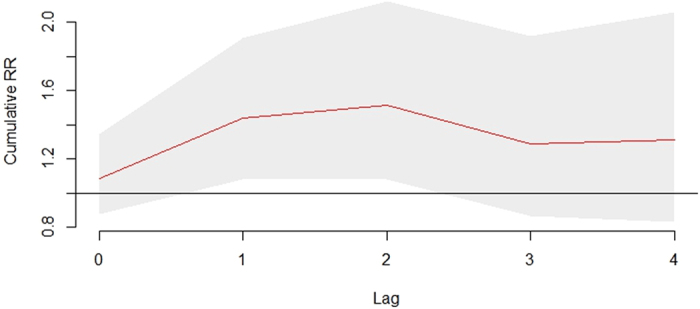
The cumulative effects of floods on bacillary dysentery in Huaihua, China from 2005 to 2011.

The risk ratios (RRs) of floods on the risk of bacillary dysentery from the DLNM model were presented in Table 2. Results showed that bacillary dysentery was associated with floods significantly at lag 1 (RR = 1.32, 95% CI: 1.12–1.56), but there was no significant association between bacillary dysentery and floods at other lag periods. The cumulative effects of floods on bacillary dysentery were presented in Fig. 3. After controlling for precipitation, temperature, relative humidity, seasonality and long-term trends, floods were associated with bacillary dysentery significantly with a cumulative RR value at lag 0–2 weeks equal to 1.52 (95% CI: 1.08–2.12). The effect of floods on bacillary dysentery at lag 1 was similar for females (RR = 1.32, 95% CI: 1.06–1.65) and males (RR = 1.30, 95% CI: 1.06–1.61). But the cumulative effects at lag 0–2 weeks were significant in females (RR = 1.65, 95% CI: 1.06–2.56), while not in males (RR = 1.35, 95% CI: 0.88–2.07). The most vulnerable age group was 15–64 years old group (RR at lag 1 = 1.39, 95% CI: 1.12–1.72). Farmers appeared to be more vulnerable than workers, children, students and other occupations.

### Sensitivity analyses

Sensitivity analyses were conducted to check whether our coefficient estimates were robust. The effects changed little when using the full-year data instead of the data in flood season (Supplementary Figure S1). When changing df (2–8) for precipitation, relative humidity, time and week of year, we found that the effect estimates at lag 1 period did not change substantially (Supplementary Figure S2). Similar effects of floods on bacillary dysentery were observed when using different models (e.g. single lag model, unconstrained DLNM model, and constrained DLNM model) (Supplementary Figure S3). Supplementary Figure S4 shows that model residuals were independent over time with a normal distribution. Supplementary Figure S5 is the ACF and PACF plots of residuals, which showed that there was no apparent autocorrelation of model residuals.

## Discussion

In recent years, bacillary dysentery has been recognized as a significant infectious disease related to climate change. Our study has quantified the lagged and cumulative effects of floods on the risk of bacillary dysentery in Huaihua, China using a distributed lag non-linear model. After controlling for the meteorological factors, seasonality and long-term trend, results indicate that floods may play an important role in the epidemic of bacillary dysentery. Although this study is based on Huaihua city only, the real impact of bacillary dysentery due to floods might be much greater, given the large population at risk and frequent floods in China. From 2000–2010 floods has affected more than 1.7 billion people in China. The direct economic losses caused by floods was approximately 1.39 trillion yuan. People at risk of floods may increase due to the rapid urbanization in China^13^. The results from this study might be applicable to most city in south and east China, because climates in those places were similar with that in Hunan.

An increased risk of diarrheal disease following floods has been reported all over the world. During the 1993 flooding in Brazil, the flooding was significantly associated with diarrhea^9^. A German study also showed that the major risk factor for diarrhea was contacting with floodwater^14^. A study from Anhui of China indicated floods were significantly associated with an increased risk of infectious diarrhea^15^. However, limited number of studies have examined the effects of floods on bacillary dysentery^10^,^16^.

In our study, results of the DLNM show that floods were associated with an increased risk of bacillary dysentery with 3 weeks’ effects after adjustment for meteorological factors, seasonality and long-term trend. However, the underlying mechanisms by which floods influences the bacillary dysentery are not yet clear. The probability of ingesting water or food contaminated by *Shigella* is likely to increase during floods. A study in Pakistan indicated that twenty percent of the drinking water samples collected during flood period were contaminated with *Shigella* and other enter pathogens including *Vibrio cholera*, *Salmonella*, *Staphylococcus aureus* and others^17^. Other studies also showed that contamination of drinking water was associated with water-borne disease outbreaks such as dysentery, cholera, hepatitis A, typhoid fever, and other gastrointestinal diseases after floods^18^,^19^.

Continuous precipitation during floods can mobilize pathogens and transport them into the aquatic environment, increasing the microbiological agents on surface water^19^. Floods can also destroy the sewage systems and waste-disposal systems, washing contaminants into drinking water. These may cause the local water quality seriously deteriorated and lead to a lack of clean water and food supply. As a result, the transmission of enteric pathogens and communicable diseases may increase during floods^20^. A study in Bangladesh also showed the flooding-induced breakdown of sanitary conditions is likely the principal mediator of the effect of climate on the infectious disease^21^.

Our study also found that the effect of floods on bacillary dysentery in the group of 15–64 was significant. This may because that people aged 15–64 years old participated in more relief work and engaged the reconstruction work more frequently than the other groups, leading to a higher exposure in the flood period. Farmers appeared to be more vulnerable than workers, children, students and other occupations. Kirsch *et al*. also demonstrates significantly worse impact and a slower recovery for rural area after the flood^22^. A possible reason is that rural areas usually have poor sanitation and medical conditions. Farmers usually do not have access to a flush toilet and probably not to clean water after floods, which may increase the risk of bacillary dysentery. Davis *et al*. found that rural communities are faced with a myriad of health care disparities, each posing as a barrier to timely response and complete recovery from a disaster, including insufficient public health infrastructure and disproportionate access to adequate medical care^23^.

It is not clear whether the difference between genders was caused by different behaviors to flood responses. A possible explanation is that during floods females may not only participated in the reconstruction work, but also prepare the food and water, care for the elderly and children. Psychological stress from increased responsibilities may cause fatigue and increased vulnerability to diseases during and after floods. Lowe *et al*. also found that the psychological and physiological health effects of floods appear disproportionately borne by females, elderly and children during floods^24^.

Urban areas around the world have expanded rapidly in recent years^25^. Nearly half of the urban expansion is projected to take place in Asia, especially in China which was highly prone to flooding from rivers and coastal surge^26^. Rapid urbanization in China has an adverse impact on urban hydrological processes, particularly in increasing the urban flood risks^13^. A study in Indonesia showed that urban expansion drives large increases in flood risk^27^. In the rapidly urbanized China cities, urban floods may cause health risks such as breaks out of infectious diseases by causing sewer water overflow and flushing foul water to public area, especially for the immigrant rural workers with poor sanitation and health services.

Limitations of our study should be acknowledged. Firstly, some confounding factors, such as the degree of flood disaster and different population immune levels could not be included in our study. Secondly, we could only obtain the weekly disease data while the daily data may be a better choice for the analysis of lag effects. Thirdly, we only focused on one city. The results might not be generalizable to other areas, particularly for those places with different climates.

In conclusion, this study provides evidence that floods may play an important role in the epidemic of bacillary dysentery in study area. People aged 15–64 years old, females, and farmers appeared to be more vulnerable than the others. Our findings have significant implications for developing local strategies to prevent and reduce bacillary dysentery given more floods have been predicted in the future due to climate change.

## Methods

### Study area and period

**Figure 4:**
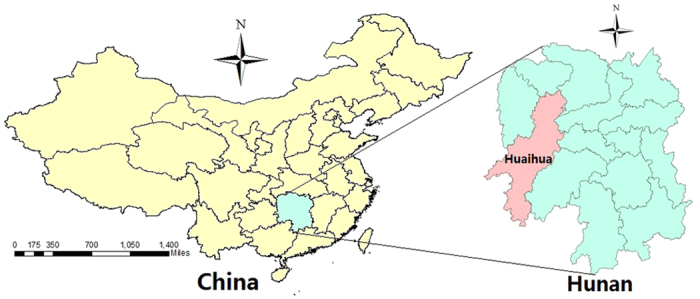
Location of the study area in Hunan province, China. The map was generated using ArcGIS 10.2 (Environmental Systems Research Institute, Redlands California, America) Available at: http://www.esri.com/software/arcgis/arcgis-for-desktop.

The study was conducted in Huaihua a city located along the Yaun River, a tributary of the Yangtze River, in the Hunan province between latitudes 25°54′ and 29°00′N and longitudes 108°48′ and 111°06′E (Fig. 4). The city is generally characterized by a subtropical humid monsoon climate with an annual average temperature of 16.4 °C and an annual average rainfall of 1600 mm. Huaihua has an area of 27,564 square kilometers and a population of 4.74 million in 2010. Given the seasonal distribution of floods and bacillary dysentery in Huaihua, periods between April and September (i.e. the flood season) from 2005–2011 were chosen as the main study periods.

### Data collection and management

#### Disease surveillance data

Weekly number of bacillary dysentery cases from 2005–2011 were obtained from the National Notifiable Disease Surveillance System (NDSS). The definition of bacillary dysentery, according to the NSDD, is a group of the human diseases that are caused by *Shigellae*, accompanied by fever, abdominal pain, tenesmus and bloody or mucus stool as the typical clinical presentation. In this study, all bacillary dysentery cases were defined based on the diagnostic criteria and principles of management for dysentery (GB 16002–1995), issued by the Ministry of Health of the People’s Republic of China^28^. Only the cases confirmed both clinically and by laboratory tests, including microscopic examination and biochemical identification, were included in our study. In China, bacillary dysentery is a statutory notifiable category B infectious disease. According to the National Communicable Disease Control Act, physicians in hospitals must report every case of bacillary dysentery to the local health authority. The local health authority must report these cases to the next level of the organization within 24 h^29^. The Direct Network Report system for infectious diseases has been established and applied well since Jan 1, 2004 in China^30^. Therefore, it is believed that the degree of compliance with disease notification over the study period was consistent.

#### Floods and meteorological data

Floods is defined as an extreme climate event with flooding and geological hazards such as debris flow, landslide after local or regional heavy rain process, it must fulfill at least one of the following criteria: (1) Ten or more people reported killed. (2) 50000 or more hectares of farmland reported damaged. (3) Cause a direct economic loss of 100 or more million Chinese yuan. Flood data was extracted from the Yearbooks of Meteorological Disasters in China, which recorded the occurrence time, number of deaths, damaged areas, and economic loss of floods^3^.

Weekly meteorological data over the same period were obtained from the China Meteorological Data Sharing Service System (http://cdc.cma.gov.cn/). The meteorological variables included weekly cumulative precipitation, weekly average temperature, and weekly average relative humidity.

### Statistical analysis

A generalized linear mixed model (GLMM) combined with a distributed lag non-linear model (DLNM) was applied to quantify the distributed lag effects of floods on bacillary dysentery, with weekly counts of bacillary dysentery as the dependent variable and floods as the independent variable adjusted for potential confounders. A quasi-Poisson regression was used to deal with the over dispersion of Poisson distribution. Generalized linear mixed model combined with distributed lag non-linear model is widely used in time series studies examining temperature, air pollution and health outcomes^31^,^32^,^33^. To control for confounder, in study design, time series analysis of diseases and extreme weather events such as heat wave or extreme precipitation event was usually conducted in a period when most extreme event occurred^34^,^35^. For our study, we chose the flood season (April-September) as our study period because almost all floods occurred in this period. The natural cubic spline used in GLMM is a flexible and effective technique for adjustment for nonlinear confounding effects of seasonality, long-term trends, and weather variables. The main advantage of DLNM is that it allows the model to describe the lag structure of exposure–response relationships, which in turn provides an estimate of the cumulative effect and delayed effect^36^.

#### Potential confounders

To control for any long term trend, we used a natural cubic spline with three degrees of freedom (df) for time. We used a natural cubic spline with four df for week of year (woy) to control for any seasonal trend^37^. Previous studies have reported that temperature, precipitation and humidity, which linked to the replication, persistence, and transmission of pathogens in the environment, were associated with diarrheal diseases^38^,^39^,^40^. Therefore, we used a smooth function of natural cubic spline with three df in DLNM for mean temperature, cumulative precipitation and relative humidity to adjust for potential effects of these meteorological factors.

#### Lags

Due to the delayed environmental transport of pathogens and delayed onset of clinical symptoms, morbidity of bacillary dysentery was expected to peak several days after the occurrence of floods. For example, a study from China reported floods can significantly increase the risk of dysentery within one month^10^. Thus, the association in our model was explored across a 4-week lag. Models which allowed for lagged exposure effects can be divide into the single lag model, unconstrained DLNM model and the constrained DLNM model. The selection of our final model was based on the distribution of lagged effects, not the index of model fitting. We chose the constrained DLNM model for two reasons. Firstly, all the lagged predictors can be simultaneously entered in the model. Secondly, after imposing some constraints on the effect estimates for the different lags, collinearity was significantly reduced. Therefore, fewer parameters need to be estimated, and associations at individual lags could be estimated with a greater precision^41^.

The main analysis was conducted with a distributed lag model to evaluate the lag effects and cumulative effects of floods on bacillary dysentery. Distributed lag models, widely utilized in air pollution studies and temperature studies, provide a systematic way to investigate the distribution of effects over time^31^,^42^,^43^. We constrained model coefficients using the lag number to fit a natural cubic spline function to reduce collinearity^36^. This model can estimate the delayed and cumulative effect of floods on the morbidity of bacillary dysentery over the entire lag period simultaneously (Model 1):

Model 1:


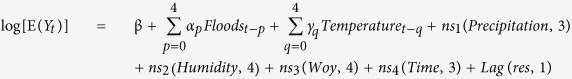


where *Y*_*t*_ denoted the weekly number of bacillary dysentery cases at time *t*. *Floods* were a categorical variable including non-flooded or flooded weeks and represented by 0 and 1, respectively. The *α*_*p*_ was the effect estimate of the floods *p* days before the day of illness. The *γ*_*q*_ was the effect estimate of temperature *q* days before the day of illness. The *ns*_*1*_*(Precipitation, 3), ns*_*2*_*(Humidity, 3), ns*_*3*_*(Woy, 4)*, and *ns*_*4*_*(Time, 3)* were natural cubic splines of weekly cumulative precipitation, weekly average relative humidity, week of year, and time, respectively, which were designed to control the effects of meteorological factor, seasonality, and long-term trend. The *Lag (res, 1)* was the first-order lagged variable of the model residual error designed to control the autocorrelation.

#### Sensitivity analysis

As effect estimates vary with different choices of model selections and parameters specification, we conducted the following sensitivity analyses:

(1) using the full-year data instead of the data in flood season; (2) varying the df (2–8) for week of year to adjust for seasonality; (3) varying the df (2–8) for time to adjust for long-term trend; (4) changing the df (2–8) for relative humidity; (5) using different methods to evaluate the estimates, single lag model, unconstrained DLNM model, and constrained DLNM model (using the lag number to fit a polynomial function)^41^.

The level of statistically significance was set at 0.05 (two-tailed). Analyses were conducted using “dlnm” package^37^ in R 3.1.3 (R Foundation for Statistical Computing, Vienna, Austria).

## Additional Information

**How to cite this article**: Liu, Z.-D. *et al*. Distributed lag effects and vulnerable groups of floods on bacillary dysentery in Huaihua, China. *Sci. Rep*. **6**, 29456; doi: 10.1038/srep29456 (2016).

## Supplementary Material

Supplementary Information

## Figures and Tables

**Table 1:** Description of weekly bacillary dysentery cases from April to September in Huaihua, China, 2005–2011.

Category	Mean ± SD	Min	P25	Median	P75	Max
Gender						
Males	11.33 ± 6.11	1	7	10	15	30
Females	8.49 ± 4.58	0	5	8	11	30
Age (years)						
0–4	6.16 ± 3.49	0	3	6	8	19
5–14	2.42 ± 2.31	0	1	2	3	15
15–64	8.87 ± 5.17	0	5	8	12	23
65+	2.37 ± 1.90	0	1	2	3	11
Occupation						
Farmers	7.50 ± 15.51	0	4	7	10	24
Students	2.33 ± 2.60	0	1	2	3	22
Children	6.58 ± 3.69	0	4	6	8	21
Works	1.83 ± 1.57	0	1	2	3	7
Others	1.58 ± 1.54	0	0	1	2	7
Total	19.62 ± 9.38	2	12	19	25	50

**Table 2:** The risk ratios of floods on bacillary dysentery at various lag weeks from the DLNM models in Huaihua, China.

Category	Lag0	Lag1	Lag2	Lag3	Lag4	Lag0–2
Total	1.09(0.88,1.35)	1.32(1.12,1.56)*	1.06(0.89,1.25)	0.85(0.71,1.02)	1.02(0.86,1.20)	1.52(1.08,2.12)*
Gender						
Males	1.00(0.75,1.31)	1.30(1.06,1.61)*	1.04(0.84,1.29)	0.84(0.67,1.05)	1.04(0.85,1.27)	1.35(0.88,2.07)
Females	1.18(0.89,1.55)	1.32(1.06,1.65)*	1.06(0.85,1.33)	0.88(0.69,1.11)	0.96(0.77,1.20)	1.65(1.06,2.56)*
Age (years)						
0–4	1.00(0.74,1.37)	1.21(0.96,1.53)	0.99(0.78,1.26)	0.85(0.66,1.09)	0.82(0.64,1.05)	1.20(0.75,1.94)
5–14	1.17(0.73,1.88	1.31(0.85,2.01)	1.07(0.72,1.60)	0.66(0.42,1.04)	1.08(0.75,1.57)	1.64(0.74,3.64)
15–64	1.03(0.78,1.37)	1.39(1.12,1.72)*	1.02(0.81,1.27)	0.84(0.66,1.05)	1.08(0.88,1.34)	1.46(0.94,2.27)
65+	0.98(0.55,1.73)	1.22(0.79,1.87)	1.35(0.90,2.01)	1.20(0.79,1.82)	1.09(0.73,1.63)	1.61(0.68,3.78)
Occupation						
Farmers	0.85(0.60,1.20)	1.42(1.11,1.82)*	1.02(0.79,1.32)	0.80(0.60,1.05)	1.07(0.83,1.37)	1.23(0.73,2.08)
Students	1.58(0.99,2.52)	1.17(0.73,1.87)	1.07(0.69,1.66)	0.71(0.44,1.14)	1.05(0.71,1.57)	1.99(0.86,4.61)
Children	0.98(0.72,1.33)	1.21(0.96,1.53)	0.97(0.77,1.23)	0.88(0.69,1.12)	0.87(0.69,1.10)	1.15(0.72,1.84)
Works	0.92(0.57,1.50)	1.07(0.72,1.58)	1.26(0.89,1.79)	0.87(0.58,1.28)	0.90(0.62,1.30)	1.25(0.58,2.67)
Others	1.59(0.91,2.81)	1.52(0.98,2.36)	1.30(0.83,2.05)	1.18(0.77,1.80)	1.28(0.84,1.97)	3.16(1.29,7.77)*

^*^p < 0.05.
