# Supplementary material for: Distributed lag effects and vulnerable groups of floods on bacillary dysentery in Huaihua, China
Source: Sci Rep. 2016 Jul 18;6:29456. doi: 10.1038/srep29456 (PMC4947917; doi:10.1038/srep29456)
Supplement: Supplementary Information [file srep29456-s1.pdf]

Supplemental Materials

Distributed lag effects and vulnerable groups of floods on bacillary dysentery in Huaihua, China

Zhi-Dong Liu[1,5,*], Jing Li[1,5,*], Ying Zhang[2], Guo-Yong Ding[3], Xin Xu[1,5], Lu Gao[1,5], Xue-Na Liu[1,5], Qi-Yong Liu[4,5], Bao-Fa Jiang[1,5]

[1] Department of Epidemiology, School of Public Health, Shandong University, Jinan, Shandong Province, People's Republic of China;

[2] School of Public Health, China Studies Centre, The University of Sydney, New South Wales, Australia.

[3] Department of Epidemiology, School of Public Health, Taishan Medical College, Taian, Shandong Province, People's Republic of China.

[4] State Key Laboratory for Infectious Disease Prevention and Control, National Institute for Communicable Disease Control and Prevention, China CDC, Beijing 102206, PR China.

[5] Shandong University Climate Change and Health Center, Jinan, Shandong Province, People's Republic of China.

* These authors contributed equally to this work.

Correspondence and requests for materials should be addressed to B.F.J.

(bjiang@sdu.edu.cn)

OUTLINE

Title	Page
**Figure S1:** Assessing the effects of floods on bacillary dysentery using Constrained DLNM model for the full-year data.	**2**
**Figure S2:** The coefficient estimates of floods on bacillary dysentery at lag 1 when changing the df (2–8) for precipitation (A), relative humidity (B), time (C) and week of year (D).	**3**
**Figure S3:** Assessing the effects of floods on bacillary dysentery using single lag model, unconstrained DLNM model, and constrained DLNM model.	**4**
**Figure S4:** Histogram of residuals (A) and Scatter plot of residuals (B) for DLNM model.	**5**
**Figure S5:** Plots of Auto-correlation function (A) and partial auto-correlation function (B) for residuals of DLNM model.	**6**

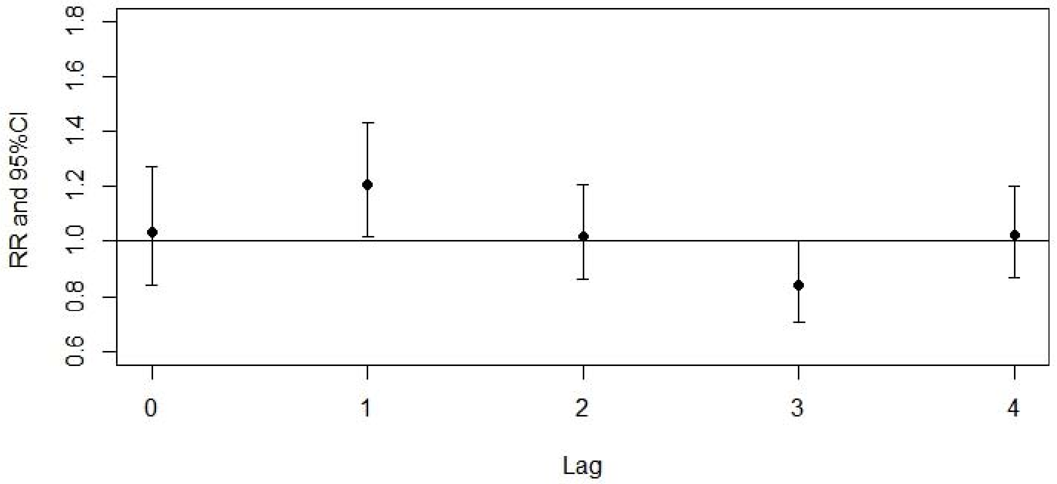

Figure S1: Assessing the effects of floods on bacillary dysentery using Constrained DLNM model for the full-year data.

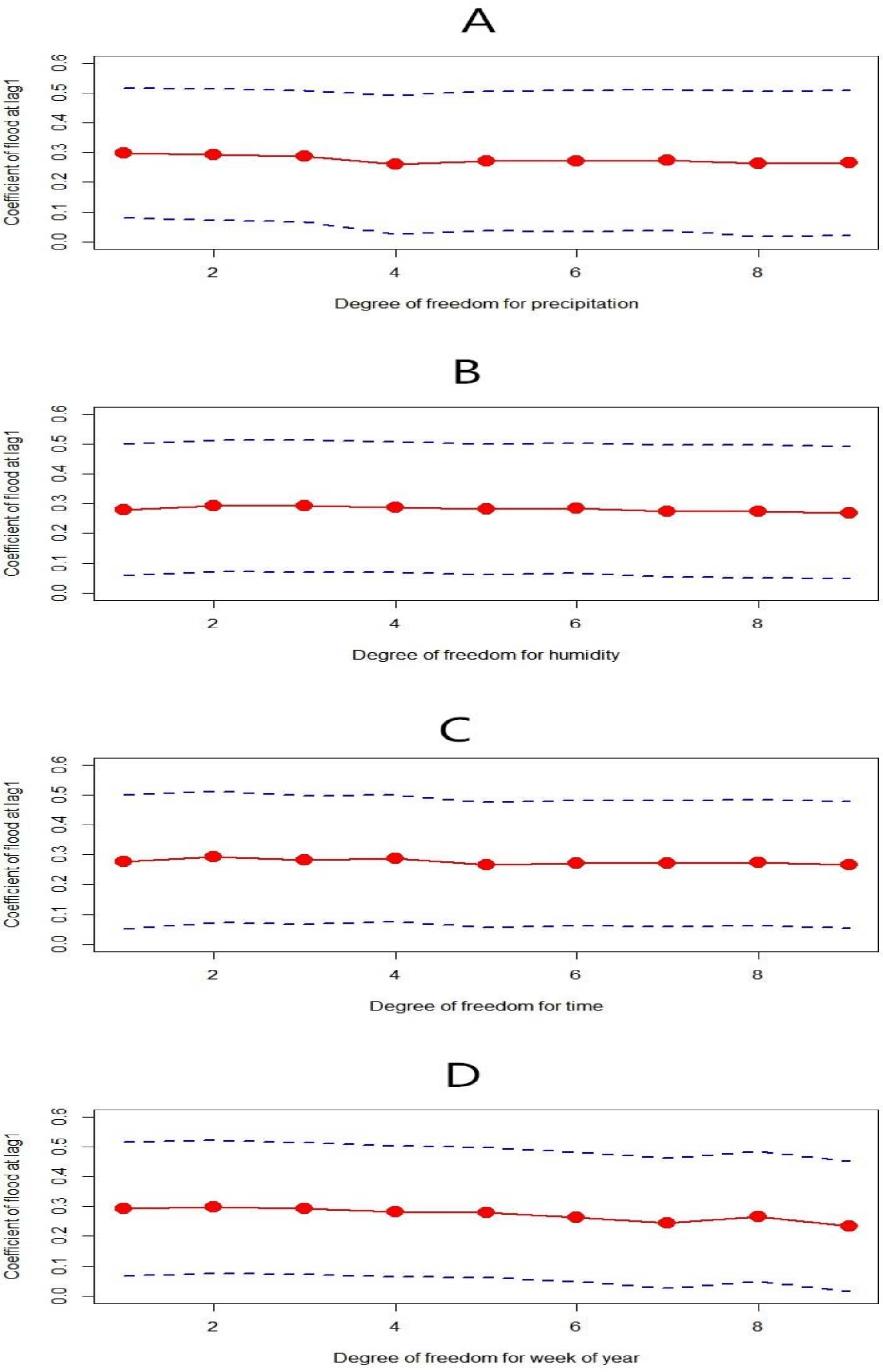

Figure S2: The coefficient estimates of floods on bacillary dysentery at lag 1 when changing the df (2–8) for precipitation (A), relative humidity (B), time (C) and week of year (D).

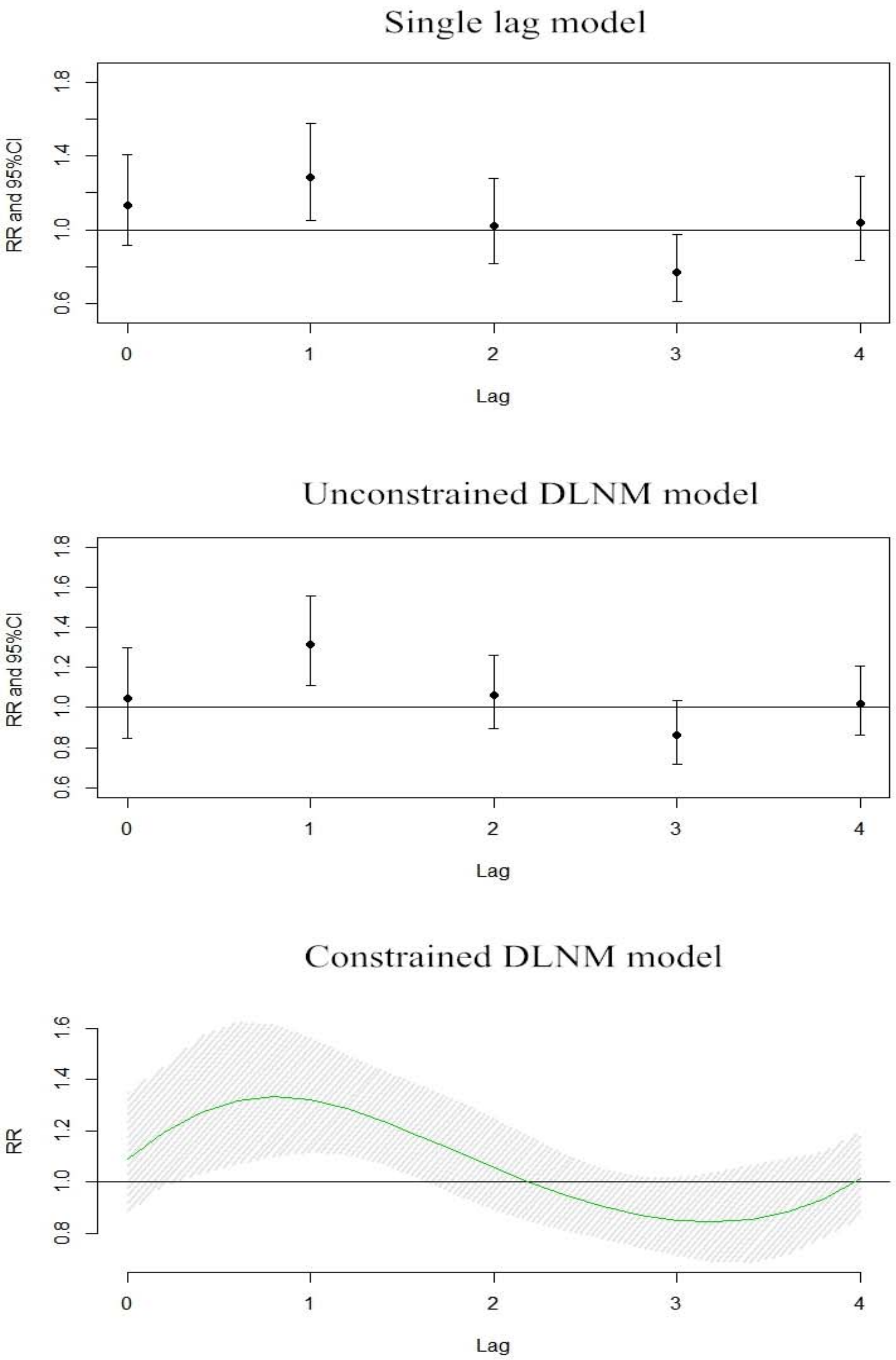

Figure S3: Assessing the effects of floods on bacillary dysentery using single lag model , Unconstrained DLNM model , and Constrained DLNM model .

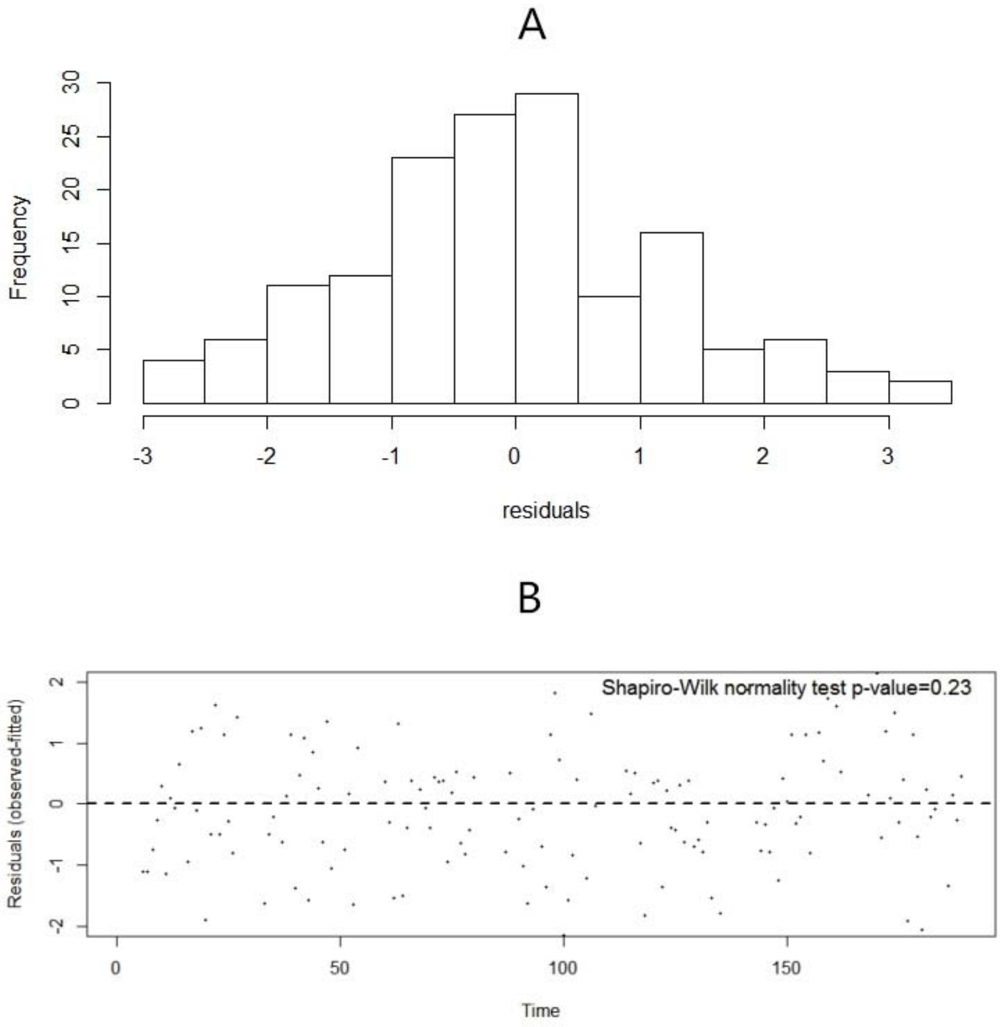

Figure S4: Histogram of residuals (A) and Scatter plot of residuals (B) for DLNM model.

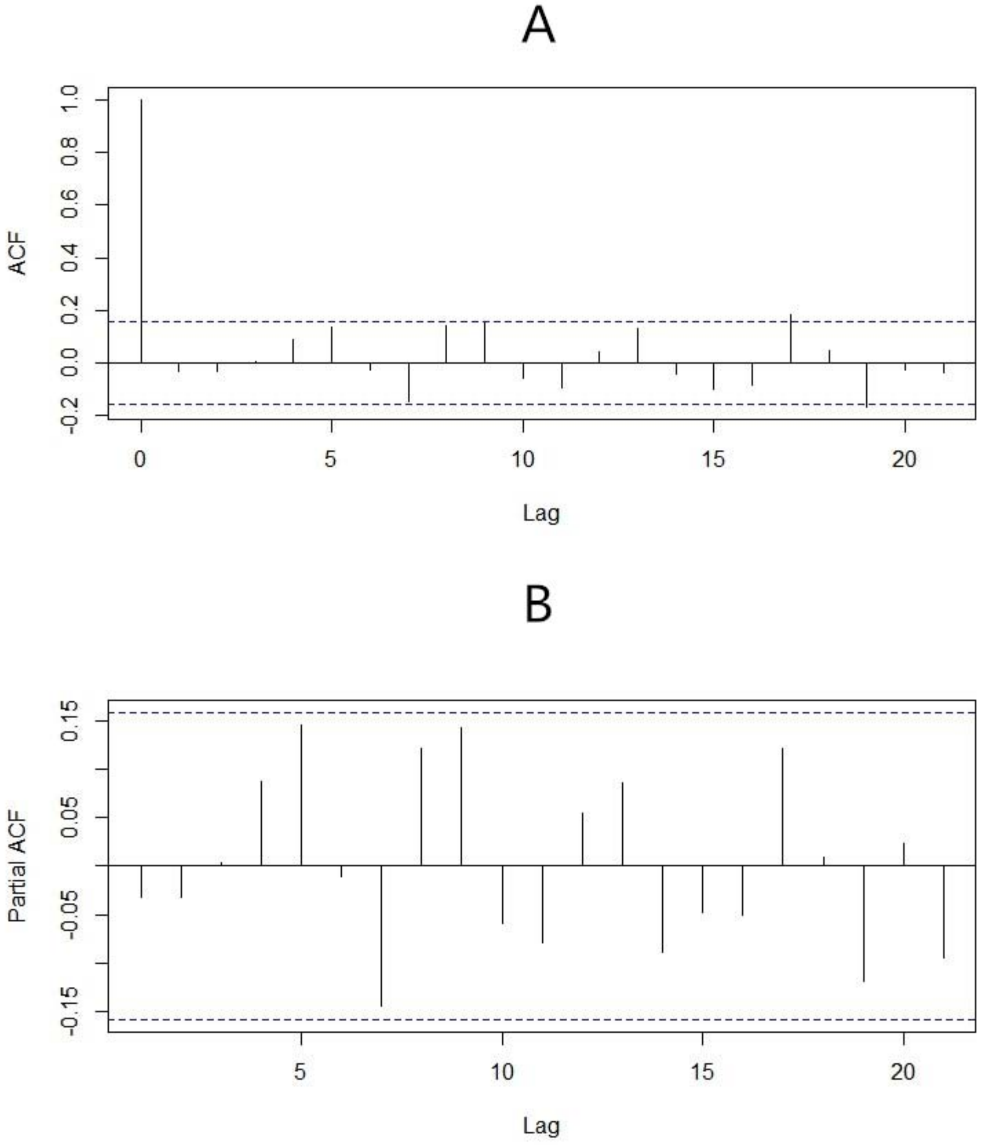

Figure S5: Plots of Auto-correlation function (A) and partial auto-correlation function (B) for residuals of DLNM model.
